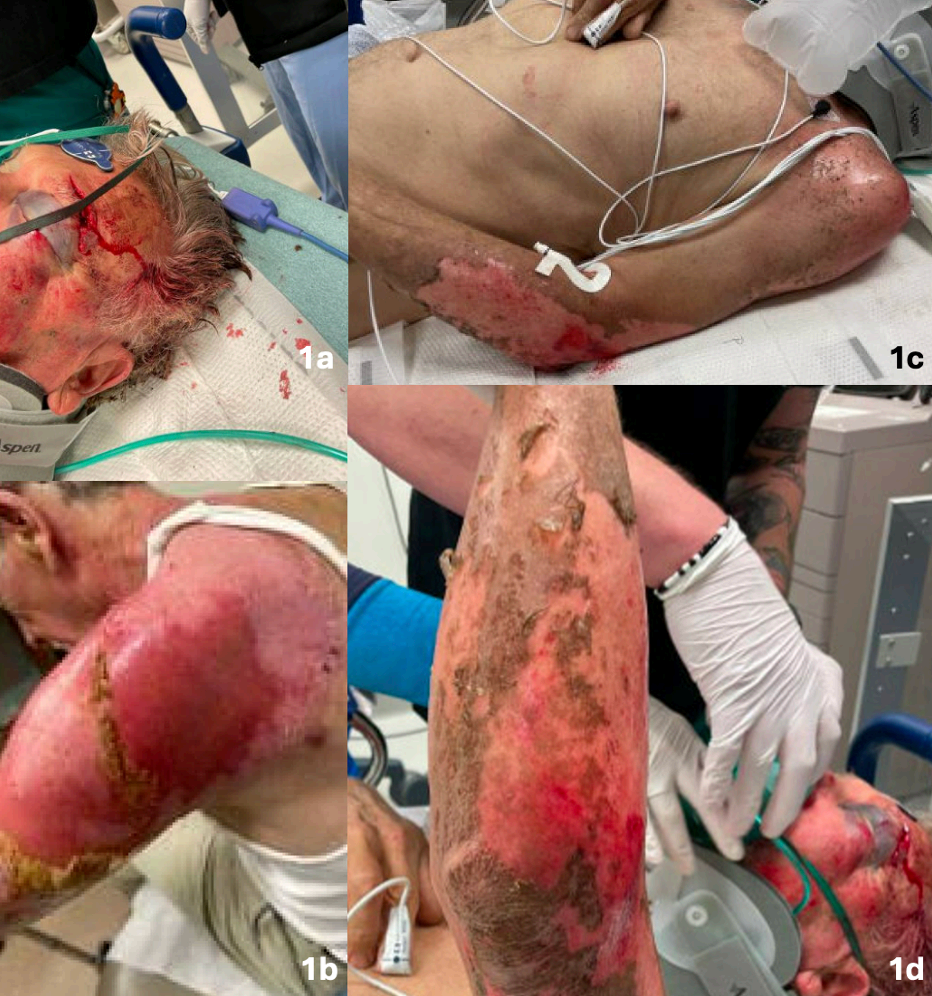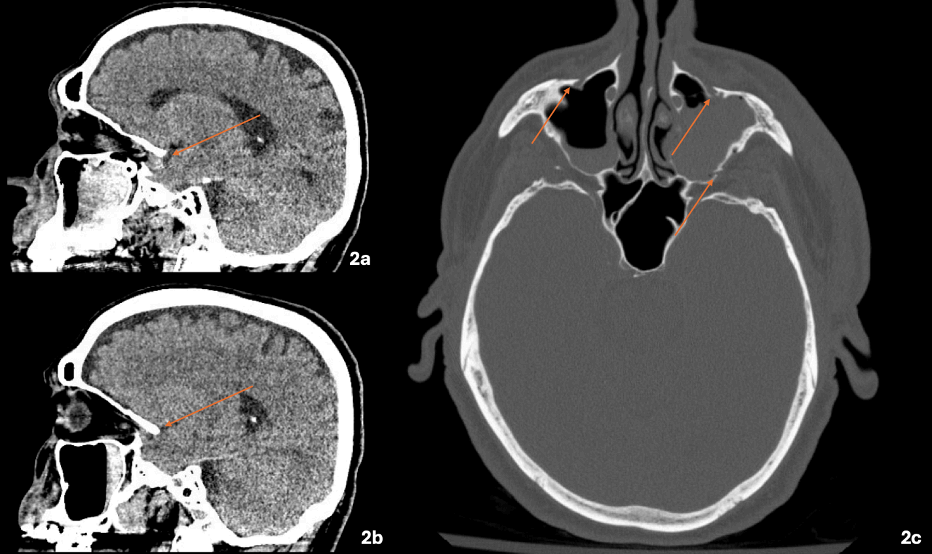# 857 The Lightning Le Fort: Contemporary Management of Lightning Strike Injuries Including Craniofacial Fractures

**DOI:** 10.1093/jbcr/iraf019.388

**Published:** 2025-04-01

**Authors:** Orr Shauly, Bo Hyun Kong, Nicholson Brant, Troy Marxen, Laura Johnson, Yuk Ming Liu, Lauren Nosanov

**Affiliations:** Emory University; Emory University School of Medicine; Emory University; Emory University School of Medicine; Walter L. Ingram Burn Center at Grady Memorial Hospital; Walter L. Ingram Burn Center at Grady Memorial Hospital; Emory University School of Medicine

## Abstract

**Introduction:**

Lightning strikes are a rare cause of multisystem trauma, often leading to complex injuries. Given the rarity of this mechanism, case reports may be instructional. Optimal care requires a multidisciplinary approach in managing such complex trauma.

**Methods:**

A 70-year-old male presented with concurrent traumatic and burn injuries following a lightning strike. Upon presentation, the patient underwent immediate stabilization according to Advanced Trauma Life Support (ATLS) and Advanced Burn Life Support (ABLS) protocols, including primary and secondary surveys. A comprehensive trauma assessment was conducted, utilizing imaging studies to determine the full extent of craniofacial and associated injuries. Imaging revealed bilateral Le Fort II and III-like fractures with intact pterygoid plates, which directed the decision towards non-operative management of the craniofacial injuries. Wound assessment revealed 6% TBSA second-degree burns on the left upper extremity which were surgically managed with debridement and the application of a biosynthetic skin substitute.

**Results:**

The patient was diagnosed with bilateral non-hemorrhagic inferior frontal lobe contusions, Le Fort II and III-like fractures, and a second-degree burn on the left upper extremity (TBSA 6%). The patient received consultations from multiple specialties, including neurosurgery, otolaryngology, ophthalmology, and plastic surgery, to develop a tailored treatment plan. The craniofacial fractures were managed non-operatively, while the burn wound was treated surgically without the need for autografting.

**Conclusions:**

This case underscores the importance of a multidisciplinary approach in managing lightning strike injuries, particularly when craniofacial fractures are involved. The unique vector of force in this patient spared the pterygoid plates, resulting in minimally displaced Le Fort-like fractures, which were managed conservatively. The use of a biosynthetic skin substitute facilitated optimal burn healing without the need for donor site creation, demonstrating its efficacy in managing partial-thickness burns.

**Applicability of Research to Practice:**

Co-location of specialized trauma and burn services along with subspecialty consultation allows for optimal, timely care of complex injuries which may occur secondary to lightning strike.

**Funding for the Study:**

N/A